# Psychotic Experiences and Overhasty Inferences Are Related to Maladaptive Learning

**DOI:** 10.1371/journal.pcbi.1005328

**Published:** 2017-01-20

**Authors:** Heiner Stuke, Hannes Stuke, Veith Andreas Weilnhammer, Katharina Schmack

**Affiliations:** 1 Department of Psychiatry and Psychotherapy, Charité–Universitätsmedizin Berlin Berlin, Germany; 2 Department of Mathematics, Freie Universität Berlin, Berlin, Germany; Technische Universitat Chemnitz, GERMANY

## Abstract

Theoretical accounts suggest that an alteration in the brain’s learning mechanisms might lead to overhasty inferences, resulting in psychotic symptoms. Here, we sought to elucidate the suggested link between maladaptive learning and psychosis. Ninety-eight healthy individuals with varying degrees of delusional ideation and hallucinatory experiences performed a probabilistic reasoning task that allowed us to quantify overhasty inferences. Replicating previous results, we found a relationship between psychotic experiences and overhasty inferences during probabilistic reasoning. Computational modelling revealed that the behavioral data was best explained by a novel computational learning model that formalizes the adaptiveness of learning by a non-linear distortion of prediction error processing, where an increased non-linearity implies a growing resilience against learning from surprising and thus unreliable information (large prediction errors). Most importantly, a decreased adaptiveness of learning predicted delusional ideation and hallucinatory experiences. Our current findings provide a formal description of the computational mechanisms underlying overhasty inferences, thereby empirically substantiating theories that link psychosis to maladaptive learning.

## Author Summary

Predictive coding theories represent a unifying account of psychosis, stating that the central psychosis-related alteration affects the interplay between prior predictions and incoming information. Since every incoming information is imprecise and potentially allows for different interpretations, prior expectations achieve the enforcement of interpretations with a higher prior probability. Disturbances in this basic framework might let unlikely interpretations come into effect, resulting in proneness for delusions and hallucinations. Here, we contribute to these theories by devising a novel computational model for behavior in a reasoning task that quantifies the participants' readiness to draw inferences from very surprising information. We thereby demonstrate that precisely this increased learning from surprising and thus potentially spurious information, as opposed to non-specific alterations in the general learning speed, predispose healthy individuals for delusions and hallucinations. The present results hence speak for the hypothesis that hallucinations and delusions arise when noisy information is considered as precise and is thus not suppressed by opposing prior beliefs. In this sense, our findings also tie with recent neurophysiological models of psychosis that posit aberrations in modulatory neurotransmitters such as dopamine (or its interactions with GABAergic interneurons) as a correlate of perturbed computations of information precision in the cortex.

## Introduction

Psychotic symptoms are a core symptom of devastating psychiatric disorders such as schizophrenia. They comprise many different kinds of experiences, among others beliefs that are unfounded in the external reality (delusions), and percepts in the absence of a causative stimulus (hallucinations). Accordingly, it poses a key challenge to theoretically and empirically establish models that can capture the multifariousness of psychotic experiences by a few (or even one) core alterations.

Influential theories [1–3] explain psychotic symptoms in the framework of predictive coding [4–6]. According to predictive coding, one central challenge for the brain is to draw inferences about the state of the external world from incoming information of relatively poor quality. It is stated that the brain deals with this challenge by recurring to predictive beliefs about the world. Such predictive beliefs are proposed to shape incoming information via top-down signals, thereby enabling stable and unitary inferences from imprecise and ambiguous information and constituting a protection against an over-interpretation of sporadically occurring irrelevant information. Importantly, predictive beliefs are assumed to be continuously updated by prediction errors. Such prediction errors are thought to drive learning via bottom-up signals, and to arise when predictive beliefs do not precisely match incoming information. Hence, ongoing learning in response to surprising information is thought to ensure the flexible adaptation of belief-dependent inferences.

Along these lines, psychotic symptoms can be framed as maladaptive learning that occurs if irrelevant information is considered as surprising and relevant due to altered prediction error signaling [1,7,8]. As a result, no stable and valid predictive beliefs would be built up and the brain would become susceptible to overhasty and erroneous inferences yielding delusions and hallucinations. In line with the idea that overhasty and erroneous perceptual inferences from irrelevant noise information are implicated in hallucinations and hallucination-proneness, hallucinatory experiences have been repeatedly associated with a greater tendency to perceive illusory contents in auditory noise [9,10]. Moreover, delusional ideation has been consistently linked to “jumping to conclusions” (JTC, see [11–13] for detailed meta-analyses), a cognitive reasoning bias that leads to a rash acceptance of hypotheses based on little evidence. However, it is a matter of ongoing debate, which particular cognitive alteration predisposes delusional and delusion-prone individuals for an overhasty acceptance of possible hypotheses [14–17]. With regard to the predictive coding account of psychosis outlined above, we suggest that JTC might reflect a pivotal alteration underlying psychotic symptoms, namely maladaptive learning from irrelevant information, leading to overhasty inferences.

To empirically test the claim that maladaptive learning contributes to psychotic symptoms, one will necessarily have to tackle the question of what constitutes adaptive learning, or, in other words, how *non-psychotic* individuals can generate and adapt beliefs sufficiently quickly in response to relevant information, and, nevertheless, resist inadequate belief revision due to irrelevant noise. Common computational learning models (e.g., [18]) formalize learning in terms of prediction errors and learning rates. Here, the current belief is obtained as a function of the prediction error that denotes the difference between the expectation (i.e., the belief before the actual observation) and the actual observation. The magnitude of this prediction error multiplied with a subject-specific learning rate determine the degree to which the belief is updated (i.e., the learning). An alternative formulation of evidence accumulation (and state estimation) calls on Bayesian filtering schemes as metaphors for neuronal computations. These schemes accumulate evidence for hidden states of the world in proportion to their estimated precision or reliability. The most celebrated Bayesian filter is called the Kalman filter, where the Kalman gain corresponds to the relative precision (inverse variance) of sensory evidence in relation to prior beliefs. Biologically plausible implementations of Kalman filtering include predictive coding, where Bayesian belief updating (i.e., evidence accumulation) is mediated by precision weighted prediction errors. In short, Rescorla Wagner models, Bayesian filtering and predictive coding are all equivalent formulations of evidence accumulation (see [19]). They all speak to the importance of precision as learning rates in modulating the impact of prediction errors on belief updating, which we will refer to as adaptive learning.

**Fig 1:**
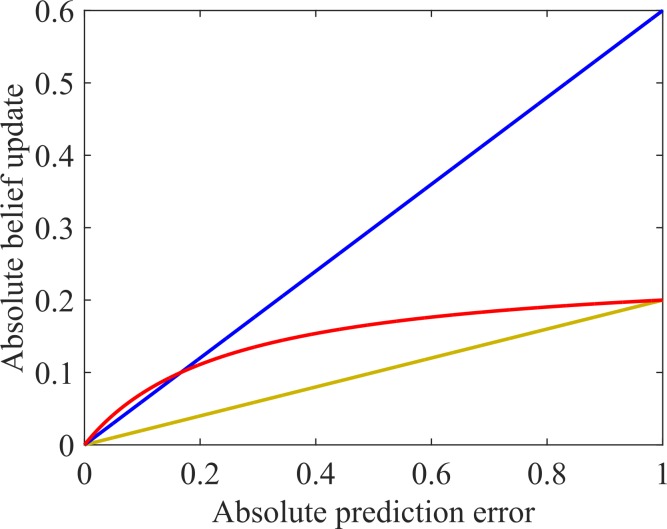
Relationship between prediction error (x-axis) and belief update (y-axis). Linear relationships with a high learning rate in blue and with low learning rate in yellow, non-linear relationship in red. We can see that although achieving a resilience against irrelevant information (attenuation of high prediction errors) comparable to the slow-learning agent in yellow, the non-linear red agent learns from small prediction errors similarly to the fast learning blue agent. Two hypotheses regarding the learning alterations that lead to hasty inferences and psychotic experiences may be suggested: Firstly, increased psychosis-proneness might be linked to a generally increased learning speed that predisposes for unfounded cognitive and perceptual inferences. According to this hypothesis, a psychosis-prone individual would behave like the blue as compared to the yellow agent (i.e., show an increased learning rate). Secondly, psychosis-proneness might be linked to a specifically decreased attenuation of large prediction errors (that can be interpreted as a reduced resilience against irrelevant and strongly surprising noise information). According to this hypothesis, a psychosis-prone individual would behave like the blue as compared to the red agent (i.e., show a decreased non-linearity of the relationship between prediction error and learning).

Thus, these common computational learning models capture adaptive learning, as opposed to maladaptive learning from irrelevant information that lead to overhasty and erroneous inferences, by small learning rates. Hence, resilience against irrelevant information would be formalized by smaller learning rates and thus comes at the expense of a generally decreased speed of learning (see Fig 1). Here, we propose a novel computational learning model that is able to capture the resilience against irrelevant information without substantially impairing the general speed of learning. The central and very simple idea of our model is that prediction errors are processed in a non-linear fashion. Concretely, we introduce a saturating non-linear function of prediction error that attenuates the effect of very large prediction errors on belief updating, relative to smaller prediction errors. Effectively, this means that very surprising or large prediction errors are treated as imprecise information; very much in the same way that we discard outliers in statistical analyses of data. In the technical literature this is known as Winsorizing and represents one of the simplest and most fundamental modifications of linear predictive coding. Formally, this compressive non-linearity can be considered a hyperprior that certain prediction errors are generated by a class of outliers that can be construed as "irrelevant". In other words, the non-linearity enables the accumulation of evidence in a way that is resistant to the effect of spurious (i.e., very surprising) events. Importantly, learning from small prediction errors is preserved, leading to adaptive inferences in response to moderately surprising and hence relevant information. Thus, our model captures the resilience against irrelevant information, and hence overhasty and erroneous inferences, by the non-linearity of prediction error processing (see Fig 1). Conversely, we would predict that a weaker resilience against irrelevant information that leads to overhasty and erroneous inferences in psychotic and psychosis-prone individuals is paralleled by a more linear processing of prediction errors.

In this work, we sought to devise a formal approach to assess and quantify the maladaptive learning mechanisms underlying overhasty and erroneous inferences related to psychotic symptoms. To this end, we devised an adapted probabilistic reasoning task that allowed us to continuously track participants’ belief trajectories. We then used this task to quantify overhasty inferences in a sample of healthy individuals with varying degrees of delusional ideation and hallucinatory experiences, based on the view that clinically relevant psychotic symptoms represent an extreme of a trait continuously distributed in the general population [20,21]. In order to investigate the computational mechanisms underlying psychosis-related biases in learning and inference, we fitted the behavioral data with our novel learning model that quantifies the adaptiveness of learning by a non-linear prediction error processing. We hypothesized that psychosis-related experiences would inversely relate to the resilience against irrelevant information quantified by the non-linearity of prediction error processing.

## Methods

### Participants and psychometric assessments

Ninety-eight healthy individuals from the general population were recruited for study participation through advertising. The study was approved by the Ethical Committee of the Charité, Universitätsmedizin Berlin. Participants who received treatment due to psychiatric diseases were excluded. After complete description of the study to the participants, written informed consent was obtained in accordance with the Declaration of Helsinki of 1975 before participation.

The participants' tendency towards delusional ideation was quantified using the Peters Delusion Inventory (PDI, [22]). The 40 items of this self-rating questionnaire cover a wide range of delusional convictions, including beliefs in the paranormal, grandiosity ideas or suspicious thoughts. For every endorsed belief, the questionnaire asks for dimensional ratings on the degree of belief-related distress, preoccupation and conviction. The total score obtained by adding up these three dimensional ratings was used for analyses.

Additionally, proneness to hallucinatory experiences was assessed with the Cardiff anomalous perception scale (CAPS, [23]). This 32-item self-rating scale assesses anomalous perceptual experiences in different sensory domains like proprioception, time perception, somatosensation and visual and auditory perception. The intensity of every anomalous perception is quantified from one to five on subscales for intrusiveness, frequency and distress. Again, the total score was calculated by adding up all subscore ratings and used for analyses.

### Probabilistic reasoning task

An adapted version of the “beads task” [24] was used to assess psychosis-related alterations in probabilistic reasoning, especially overhasty inferences such as the JTC bias. In the beads task, beads are continuously drawn from one of two different urns that contain different numerical proportions of different kinds of beads. The participants have to infer from which urn beads are currently being drawn based on their knowledge about the numerical proportions of different kinds of beads in the two urns and the number of already drawn beads of each kind. The task thus implies a continuous update of the belief about the correct urn with every new draw, which can be either consistent with the current belief about the correct urn (relevant information) or inconsistent with it (irrelevant information).

In our version of this task, the participants were shown pictures of two different lakes (a “mountain lake” and a “flatland lake”) and told that these lakes are home to a different proportion of carps and trouts with the mountain lake containing 70% carps and 30% trouts and the flatland lake 30% carps and 70% trouts. For reasons of simplicity, we will refer to the mountain lake as the "carp lake" and to the flatland lake as the "trout lake" in the following.

The task was structured in 30 rounds with a varying number of draws. On each round, fishes were sequentially angled from one of the two lakes and the participants were instructed to evaluate from which of the lakes the fishes were more likely angled in this round using the number of so far angled carps and trouts and their knowledge about the numerical proportion of fishes in the two lakes (thus with every angled carp making the carp lake more probable and every angled trout making the trout lake more probable). Moreover, participants were told that both lakes contained so many carps and trouts that the numerical proportions did not change due to the fishing.

**Fig 2:**
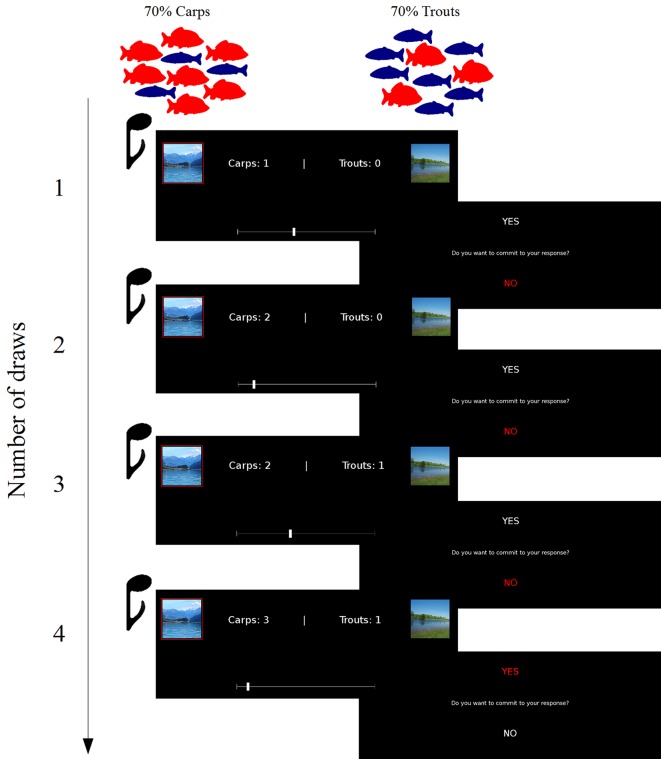
Experimental sequence of the probabilistic reasoning task. To determine, from which of the two possible lakes fishes were being angled, the participants used their knowledge about the numerical proportions of fishes in the lakes (70% carps and 30% trouts in the carp lake and vice versa in the trout lake) and the number of so far angled carps and trouts. Prior information about the lakes' probabilities was given by a tone that was–dependent on the pitch–in 80% of the cases associated with the one or the other lake. After each draw, the current belief about the correct lake was indicated on a continuous response bar. Subsequently, the participants decided if they want to make a final decision on the lake (commit to their response) or if they want to gain further information in the form of newly angled fishes (i.e., making a new draw).

Each round started with only one angled fish and, accordingly, with a rather imprecise information about which of the two lakes being correct in this round. To gain further information, participants were allowed to make new draws until they felt confident enough to make a final decision about the correct lake in this round (Fig 2). With every new draw, one new fish was angled and the number of so far angled trouts and carps was updated and presented. After each draw, the participants indicated their new belief about from which lake the fishes were probably angled in this round. For this purpose, they entered their guess and its certainty using the mouse on a visual scale (ranging from absolute certainty of the carp lake being correct at the very left to absolute certainty of the trout lake being correct at the very right with positions close to the center indicating uncertainty). In this way, we obtained a continuous assessment on the participants' current belief for each draw. After having placed their guess, the participants were asked if they wanted to commit themselves to the given response on the correct lake (by pressing either the up or the down arrow key). If they did not commit to their response, a new fish was angled (new draw). If they committed to their response, a final decision on the lake was made and a new round started with once again only one angled fish and accumulating evidence with every further draw.

To induce prediction errors even in rounds with few draws, we added a prior information about the lakes' probabilities in the form of a high- or low-pitched tone that was played shortly before every newly angled fish. In one round, always the same tone pitch was played. If the fishes were angled from the carp lake in the round, the high-pitched tone was played more frequently (80%) and if the fishes were angled from the trout lake, the low-pitched tone was played more frequently (80%). Thus, the tone pitch constituted a probabilistic initial information about the lake probabilities for each round. The associations between tone pitch and lake probabilities were learned in a preceding learning run of 15 rounds and did not change throughout the experiment. By these means, we could assess prediction errors already in the first draw (one angled fish) and increase the variance of prediction error values occurring throughout the course of the experiment.

### Relationship between jumping-to-conclusions, delusional convictions and anomalous perceptions

To quantify the tendency towards overhasty inferences in each participant, we calculated the mean number of draws a participant needed on each round before committing to a final decision. This measure ("draws to decision") is an accepted measure for the JTC bias found to be associated with psychotic symptoms [14].

To replicate prior findings that participants with growing psychosis proneness tend to exert jumping-to-conclusions (see introduction), we tested associations between the participants’ draws to decision and the tendency towards delusional convictions (PDI scores) as well as the proneness to hallucinatory experiences (CAPS scores) in two different ways. Firstly, as suggested by [25], we performed a binary analysis with our sample separated into two groups (with and without JTC). There were only six of 94 participants showing JTC according to the commonly applied threshold of two draws to decision, probably due to the differing set-up of our adapted version of the lake task (introduction of prior knowledge associated with the tone, usage of continuous response bar). Thus, we used a slightly higher threshold and considered participants in the lowest quartile of draws to decision (i.e., with an average of 3.2 or less draws to decision) as exhibiting JTC and compared their PDI and CAPS scores with the remaining (non-JTC) sample. Secondly, we investigated continuous relationships by correlating PDI and CAPS scores with the mean number of draws to decision.

Since the distribution of PDI and CAPS scores differed significantly from a normal distribution (Z = 1.374, p = 0.046 for PDI scores and Z = 1.941, p = 0.001 for CAPS scores, one-sample Kolmogorov-Smirnov tests), we used non-parametric Mann-Whitney tests for the first (categorical) and Spearman rank correlations for the second (correlational) analysis.

To our knowledge, there are no previous studies reporting associations between hallucinations and JTC, giving our analysis on relationships between CAPS scores and JTC a rather exploratory character. Nevertheless, because we tested associations between JTC and both PDI and CAPS scores, we report among uncorrected p values also p values with adjustment for multiple testing (tests for PDI and CAPS scores, e.g., two tests with correlated outcomes). To this end, p values were adjusted according to the approach proposed by [26] and outlined in [27] for multiple comparisons with correlated outcomes.

### Computational modeling

By fitting the behavioral data with computational learning models, we aimed at quantifying the resilience against irrelevant information and thereby assessing the adaptiveness of learning, which we expected to be inversely related to psychotic symptoms.

Two computational models were designed to track the participants' trajectories of belief in the probabilistic reasoning task. Firstly, we applied a conventional linear prediction-error-based learning model (e.g., [28]). Secondly, we developed a novel model that enabled the quantification of the participants’ resilience against irrelevant information through a non-linear relationship between prediction error and learning, which we expected to provide a more precise description of adaptive learning in probabilistic reasoning.

In both models, the participants’ beliefs about the correct lake were captured on a trial-by-trial basis as a continuous value between 0 (certainty that the “carp lake” is correct) and 1 (certainty that the “trout lake” is correct). Thus, the high-pitched tone as well as newly angled carps brought the belief nearer to the 0 and the low-pitched tone as well as newly angled trouts nearer to the 1. Eq 1 shows accordingly, that the neutral belief of 0.5 was initially shifted towards 1 in case of the "trout-lake"-associated low-pitched tone and towards 0 in case of the "carp-lake"-associated high-pitched tone and that the magnitude of the tone-dependent belief shift depended on the subject-specific parameter θ. Since the neutral belief of 0.5 could be shifted by maximally 0.5 by the tone, we used a uniform distribution between 0 and 0.5 as a prior distribution for the estimation of θ values based upon choice behavior.

$$\begin{array}{l} Initial\;ton\mathrm{e-d}ependent\;belief\;(\pm :\;+\;if\;trout\;is\;angled,\;\text{-}\;if\;carp\;is\;angled).\\ b_{1}=0.5+\theta \end{array}$$ Eq1

Whereas this initial tone-dependent belief was calculated in the same way in both models, the effect of newly angled fishes differed between the conventional linear and our novel non-linear model. In the linear model, the prediction error determined the learning linearly. Eq 2 shows that the belief update here depended on the non-modified prediction error b_i-1_ –o_i_ (difference between the former belief b_i-1_ and the current observation o_i_) that was multiplied with a subject-specific constant learning rate α that captures the general rapidity of belief generation regardless of the typicality of the new information. Since the learning rate is naturally bounded between 0 and 1, we used a uniform distribution between 0 and 1 as a prior distribution for estimation of α values based upon choice behavior.

$$\begin{array}{l} Linear\;belief\;update\;(bi:\;current\;belief,\;oi:\;current\;(binary)\;observation\;(1\;if\;trout\;is\;angled,\;0\;if\;carp\;is\;angled),\;\pm :\;+\;if\;trout\;is\;angled,\;\text{-}\;if\;carp\;is\;angled).\\ b_{i}=b_{i-1}\pm \alpha *\left(b_{i-1}-o_{i}\right) \end{array}$$ Eq2

In the non-linear model on the other hand, the learning depended on the prediction error with a varying degree of non-linearity expressed by the non-linearity parameter ζ. Please note that high values of ζ imply a marked non-linearity / flattening of the relationship between prediction error and learning, whereas this relationship is linear for ζ = 0. Thus, high values of ζ imply a strong resilience against irrelevant information, since high prediction errors have a reduced impact on learning in this case: Hence, this modulation can be thought of as a dynamic learning rate that adaptively decreases if information is unreliable and potentially irrelevant. As in common behavioral learning models, the resulting non-linear learning term was multiplied with a subject-specific constant learning rate α that captures the general rapidity of belief generation regardless of the typicality of the new information. Eq 3 shows how the current belief b_i_ is updated depending on the learning rate α and the prediction error b_i-1_ –o_i_, whose impact on the learning decreases with increasing values of ζ. Compared to other possible implementations of a non-linear prediction error, the definition outlined above has the advantage of yielding one simple parameter that determines the degree of non-linearity and is zero for an entirely linear relationship between prediction error and learning. Furthermore and importantly, it cannot generate overshooting beliefs below zero or above one without having to assume an additional softmax transformation (see proof in the Supplementary Material). Fig 1 shows exemplary relationships between prediction error and learning, with linear relationships (ζ = 0) in blue (α = 0.6) and yellow (α = 0.2) and a non-linear relationship in red with ζ = 4 and α = 1. Since such a non-linear prediction error processing has to our knowledge not been implemented so far, we used a uniform distribution between 0 and 5 (thus allowing for a wide range of non-linearity) as a prior distribution for estimation of ζ values based upon choice behavior.

$$\begin{array}{l} No\mathrm{n-l}inear\;belief\;update\;(bi:\;current\;belief,\;oi:\;current\;(binary)\;observation\;(1\;if\;trout\;is\;angled,\;0\;if\;carp\;is\;angled),\;\pm :\;+\;if\;trout\;is\;angled,\;\text{-}\;if\;carp\;is\;angled).\\ b_{i}=b_{i-1}\pm \alpha *\frac{1}{\zeta +\frac{1}{\left(b_{i-1}-o_{i}\right)}} \end{array}$$ Eq3

**Fig 3:**
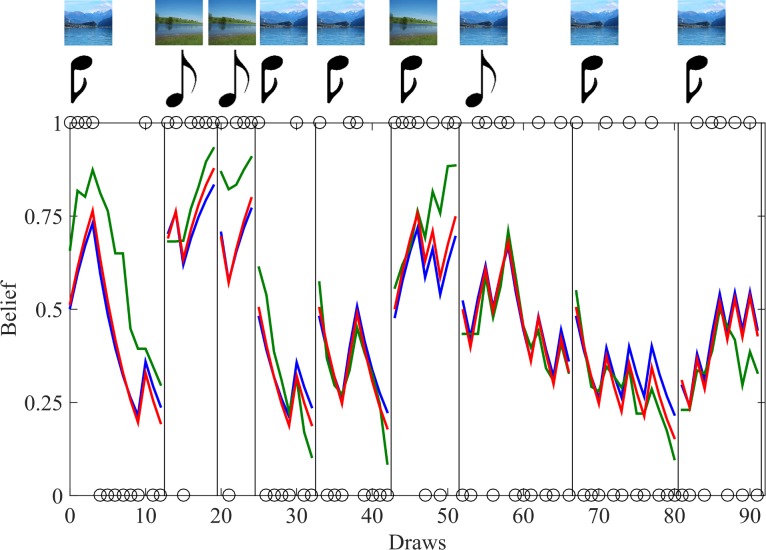
Sample trajectory of belief for nine rounds in one exemplary participant. The participant's belief, i.e., the probability with which the participant considered the one or the other lake as correct (indicated on the response bar), is shown in green, the predicted belief by the non-linear model in red and by the linear model in blue. The upper bound (belief = 1) indicates absolute certainty that the trout lake is correct in this round, whereas the lower bound (belief = 0) indicates absolute certainty that the carp lake is correct. Circles on the upper bound mean that a trout and on the lower bound that a carp has been angled in this draw. Vertical lines indicate that a decision on the lake has been made and a new round started. If a decision was made at a current belief of below 0.5, the participant had decided in favor of the carp lake (and accordingly above 0.5 in favor of the trout lake). The top row shows which of the two lakes has been correct in each round and we can see that the participant gave no incorrect answer in these nine rounds. The row below shows if a high- or a low-pitched tone has been played. Thus, we can see that the sixth and seventh round had unrepresentative tone meanings.

Both models were applied to explain the trajectory of the participants' beliefs about the correct lake throughout the course of the experiment. For this purpose, the trial-by-trial belief indicated on the continuous response bar was scaled between 0 and 1, yielding the trajectory of belief vector g. Subsequently, each participants' trajectory of belief g was fitted with both models using the VBA Toolbox for Matlab [29]. This approach uses Variational Bayesian methods to estimate the parameter values of our two models for which the trajectory of the belief b predicted by the model optimally traces the real belief g indicated by the participants (Fig 3). Furthermore, the (lower bound on the) model's evidence (marginal likelihood), i.e., the likelihood that the real trajectory of belief g could have been generated by the respective model, was computed and used for model comparison (see below).

Summing up, the following parameters were estimated to optimally model the participants' behavior:

θ: Tone-dependent initial (i.e., prior) belief

α: General learning rate

ζ: Non-linear prediction error processing (resilience against irrelevant information, ζ = 0 in case of the linear model)

### Model comparison

To test if the non-linear model that allowed for a non-linear relationship between prediction error and learning explained the participants’ behavior better than the conventional linear model, we performed a formal Bayesian model comparison between the two models. Therefore, both models were used to fit the participants' continuous belief trajectories and the resulting model evidences were compared using the approach outlined in [30] and implemented in Statistical Parametric Mapping 12 (SPM 12). In this approach, the ability of a model to accurately predict the participants behavior is balanced against its complexity, where growing model complexity is punished. In our case, this means that if the non-linear model proves to be superior in the model comparison, the growing complexity which results from the inclusion of the additional non-linearity parameter is overcompensated by the gain in accuracy afforded by it. In addition to formal model comparison, we calculated the explained variance R^2^ of each participants’ belief trajectory by the model in order to obtain a clear measure of how well the models were able to capture the participants’ behavior. Please note that in contrast to Bayesian model comparison as described above, this assessment of model fit does not take into account the model complexity and is therefore not an appropriate measure for formal model comparison.

### Learning and usage of the tone parameter

The introduction of the tone as prior information about the lakes' probabilities allowed us to assess prediction errors even in participants with few draws to decision (see above). However, it has to be ensured, that the tone meanings has been learned by the participants during the learning run. Moreover, it has to be ensured, that differences in learning the tone meaning associated with varying psychosis-proneness constitute no alternative explanation for the relationships between psychosis-proneness and altered information processing assessed in this study.

For the former purpose, we performed a “proof of concept” model comparison between the full model with the tone parameter estimated as a free parameter and a model in which the tone parameter was fixed to 0 (i.e., that assumes that the tone has not been used by the participants). A superiority of the full model over the model with fixed tone parameter would prove that the tone was indeed used by the participants. Formal model comparison was carried out as described in the “model comparison” paragraph above.

For the latter purpose (excluding psychosis-related differences in learning the tone), we correlated PDI and CAPS scores with the value of the tone parameter θ. A lack of such a relationship would demonstrate that there is no evidence that learning and usage of the tone depended on the participants' psychosis proneness.

### Distribution of the non-linearity parameter

Contrary to most computational learning models, our model included a non-linear relationship between prediction error and learning that captures a reduced impact of high prediction errors resulting in adaptive reduced learning from irrelevant information, and, thus, in a resilience against overhasty and erroneous inferences. The degree of the adaptiveness of learning is quantified by the non-linearity parameter ζ, with higher values of ζ indicating a stronger adaptiveness of learning. Because there are to our knowledge no prior studies that implemented this kind of non-linear prediction error processing, we computed the subject-specific values of ζ without prior assumptions on its distribution, i.e., with a uniform prior distribution that made every value between 0 and 5 equally likely. To generally assess the form in which the degree of resilience against irrelevant information was distributed in our sample of participants, we tested the hypotheses that the estimated values of ζ were uniformly distributed (like the naive prior distribution) or normally distributed (like many psychological and biological variables). To this end, one-sample Kolmogorov-Smirnov tests were applied.

### Relationship between model parameters and jumping-to-conclusions

To test if a low resilience against irrelevant information was related to overhasty inferences (i.e., to jumping-to-conclusions), we correlated the values of ζ with the participants' mean number of draws to decision in the probabilistic reasoning task. This analysis primarily served as a proof-of-concept since the parameter values of an appropriate behavioral model for the probabilistic reasoning task should be able to explain a large part of the interindividual variance in jumping-to-conclusions.

To additionally demonstrate that JTC can be more accurately explained by a reduced resilience against irrelevant information compared to a generally increased learning speed, we subsequently calculated correlations between the number of draws to decision and the participants’ learning rates (values of α in the linear model).

### Relationships between model parameters, delusional convictions and anomalous perceptions

To test our hypothesis that psychosis-related experiences would inversely relate to the adaptiveness of learning, we calculated correlations between the participants’ values of ζ and the proneness for delusional convictions (PDI scores) as well as hallucinatory experiences (CAPS scores). Since the distribution of PDI and CAPS scores differed significantly from a normal distribution (as reported above), we again used non-parametric Spearman rank correlations for this purpose.

To additionally demonstrate that this relationship is specific for a reduced resilience against irrelevant information and does not only reflect a generally increased learning speed, we repeated the analysis including the participants’ learning rates (values of α in the linear model) as a covariate in a Spearman partial correlation between PDI and CAPS scores and ζ values.

Finally, we repeated these analyses correcting for multiple comparisons with correlated outcomes (according to [26], see above).

## Results

### Sample characteristics

Four participants were excluded from analyses because they showed excessively high error rates at the final decision of the probabilistic reasoning task (more than two standard deviations above sample mean, corresponding to more than 26.2% wrong decisions), suggesting that they did not perform properly in the task. The characteristics of the remaining sample of 94 participants are summarized in Table 1.

**Table 1:** Sample characteristics. PDI = Peters Delusions Inventory; CAPS = Cardiff Anomalous Perceptions Scale.

**Characteristic**	**Mean (SD)**
Age	30.52 (10.08)
PDI score	64.54 (57.11)
CAPS score	30.39 (35.61)
**Characteristic**	**Absolute numbers**
Sex	female: 56; male: 38
Smoking	yes: 27; no: 67
Graduation	lower secondary school: 6; higher secondary school: 24; high school: 63; missing information: 1

### Relationship between jumping-to-conclusions, delusional convictions and anomalous perceptions

A large body of evidence has linked psychosis and psychosis-proneness to a reduced number of draws to decisions in the beads task (JTC, [11,31]). To test whether we could replicate this relationship in our current sample, we related the mean number of draws to decision to PDI scores (proneness for delusional convictions) and CAPS scores (proneness for anomalous sensory experiences). Indeed, our categorical assessment revealed a significantly increased PDI in the JTC group (n = 23, median of PDI scores = 79) as compared with the no-JTC group (n = 71, median of PDI scores = 45), Mann-Whitney test with U = 585, p = 0.042, two-sided. No significant difference was found for CAPS scores (median CAPS score in JTC group = 30, median score in no-JTC group = 15, Mann-Whitney test with U = 681,5, p = 0.233).

Correlational analyses reproduced the effect for PDI scores on a trend level (fewer draws to decision with rising PDI scores, rho = -0.177, p = 0.089, two-sided Spearman rank correlation), whereas CAPS scores again showed no significant relationship (rho = -0.154, p = 0.139, two-sided Spearman rank correlation).

When adjusted for multiple comparisons with correlated outcomes, the relationship between JTC and PDI scores was still present, but failed to reach statistical significance (p adjusted = 0.051 in the categorical analysis and p adjusted = 0.108 for the correlational analysis).

### Model comparison

To test if our model that allowed for a non-linear relationship between prediction error and learning explained the participants’ behavior better than a standard linear model, we performed a Bayesian Model Comparison between the two models. Here, the non-linear model proved to be superior to the standard linear model in explaining participants’ behavior with a protected exceedance probability of 100%. The mean value of explained variance (R^2^) of the participants trajectory of belief was 0.696 for the non-linear and 0.661 for the linear model. Since this measure of accuracy does not take into account the differing model complexity, it is not suitable for directly comparing the quality of the models. It however shows that especially the non-linear model appropriately tracked the course of the participants’ belief.

### Learning and usage of the tone parameter

With two proof-of-concept analyses, we aimed at ensuring, that the tone meaning was learned by the participants during the learning run, but that psychosis-proneness was not associated with differences in learning the tone meaning.

To ensure the significance of the tone for the participants' belief updating, we performed a formal model comparison between the full model, in which the tone parameter was estimated as an individual free parameter and a model without tone parameter (i.e., with θ fixed to 0, assuming that the tone was not used by the participants). Here, the model, in which θ was freely estimated, proved to be superior with a protected exceedance probability of 99.5%. Thus, taking into account the usage of the tone significantly improved the tracking of the participants' belief trajectory.

No significant or trend-wise relationships were found between the value of the tone parameter and delusion-proneness (PDI scores, rho = 0.030, p = 0.771, two-sided Spearman correlation) or hallucination-proneness (CAPS scores, rho = 0.038, p = 0.714), not providing any evidence that individual differences in the learning of the tone might provide an alternative explanation for the found relationships between psychosis proneness and the lowered resilience against irrelevant information.

### Distribution of the non-linearity parameter

**Fig 4:**
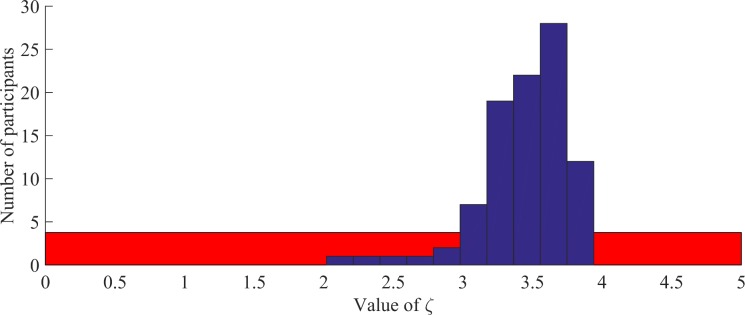
Histogram of the distribution of ζ values in our sample. Prior distribution (uniform between 0 and 5) in red, real distribution in blue. It can be seen that, contrary to the uniform prior distribution, estimated parameter values show a normal-like distribution with a mean value of 3.44. This is confirmed by Kolmogorov-Smirnov tests on the form of the underlying distribution.

The non-linearity parameter ζ quantifies the degree to which the impact of the prediction error on learning is attenuated if new information is very surprising. Accordingly, low values indicate maladaptive learning with a low resilience against irrelevant information. In common learning models, a linear relationship, i.e., a parameter value of ζ = 0, is assumed. We estimated the values of ζ that optimally explained our participants' behavior with an uninformative prior distribution uniformly distributed between 0 (linear relationship) and 5 (strongly non-linear relationship). It turned out that none of our participants showed a linear processing of the prediction error. Instead, more than 95% of the participants showed values of ζ between 2.5 and 4 with a marked peak around 3.5 (Fig 4). Interestingly, the distribution of the estimated values of ζ differed significantly from the prior uniform distribution (Z = 4.663, p < 0.001, Kolmogorov-Smirnov test), but not from a normal distribution (Z = 1.051, p = 0.219, Kolmogorov-Smirnov test). These results suggest that in our probabilistic reasoning task, learning depended on the prediction error in a non-linear fashion and all participants showed resilience against irrelevant information, although the degree of this resilience varied across participants.

### Relationships between model parameters and jumping-to-conclusions

To test if the participants’ resilience against atypical information was associated with jumping-to-conclusions behavior, we correlated parameter values of ζ with the participants mean number of draws to decision in the probabilistic reasoning task. This analysis yielded a strong positive correlation (r = 0.705, p < 0.001, Pearson Correlation), indicating that participants with a lower resilience against irrelevant information took decisions based on less evidence (JTC).

As predicted, the learning rate α of the linear model also showed a (negative) correlation with draws to decision, albeit weaker than ζ in the non-linear model (r = -0.460, p < 0.001, Pearson Correlation).

### Relationships between model parameters, delusional ideation and hallucinatory experiences

According to predictive coding models of psychosis, maladaptive learning with a reduced resilience against irrelevant information would result in overhasty and erroneous inferences, and should therefore be related to an increased proneness towards delusional ideation and hallucinatory experiences.

**Fig 5:**
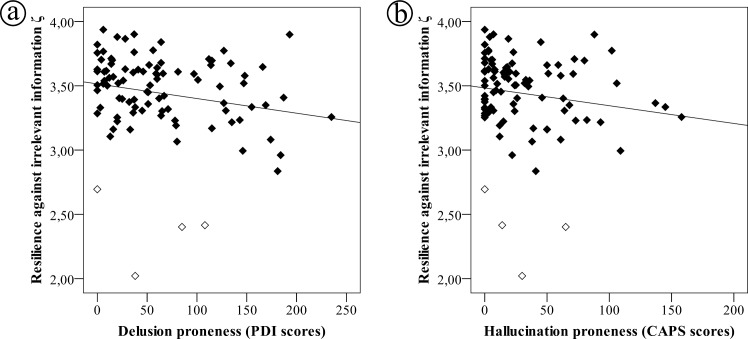
Association between resilience against irrelevant information in the probabilistic reasoning task and psychotic experiences. (a) association with delusion proneness (PDI scores, rho = -0.235, p = 0.022, Spearman’s correlation), (b) association with hallucination proneness (CAPS scores, rho = -0.198, p = 0.056). Empty squares mark outliers with ζ values two standard deviations below mean. Exclusion of these outliers yielded magnified effect sizes.

Notably and in line with this hypothesis, estimated parameter values of ζ were negatively correlated with PDI scores (rho = -0.235, p = 0.022, two-sided Spearman rank correlation, Fig 5A) and trend-wise with CAPS scores (rho = -0.198, p = 0.056, two-sided Spearman rank correlation, Fig 5B), indicating that individuals with a low resilience against irrelevant information showed an increased proneness for delusional ideation and (as a tendency) hallucinatory experiences. To exclude significant correlations due to four outliers with ζ values two standard deviations below mean (i.e., below 2.794, outliers are marked with white squares in Fig 5), we repeated these analyses excluding the outliers (thus with n = 90). This resulted in rather stronger effects (rho = -0.261, p = 0.014 for the correlation between PDI scores and ζ, rho = -0.212, p = 0.044 for the correlation between CAPS scores and ζ).

These correlations remained unchanged when controlling for the unspecific learning rate obtained from the linear model: A Spearman partial correlation including the linear learning rate as a covariate revealed similar results to those reported above (r = -0.234, p = 0.024 for correlation between PDI scores and ζ, r = -0.196, p = 0.062 for correlation between CAPS scores and ζ). This shows that effects between psychosis proneness and belief updating specifically affect the treatment of irrelevant information and cannot be explained by the nonspecific linear learning rate.

When p-values were adjusted for multiple comparisons (two tests with correlated outcomes for CAPS and PDI scores), the relationship between PDI scores and ζ values remained significant (adjusted p of two-sided Spearman rank correlation = 0.027) and the relationship between CAPS scores and ζ values a statistical trend (adjusted p of two-sided Spearman rank correlation = 0.068).

## Discussion

In the present study, we tested the claim put forward by predictive coding models [1–3] that psychotic experiences may be linked to maladaptive learning, i.e., an aberrant encoding of precision, that results from a reduced resilience against irrelevant information and leads to overhasty and erroneous inferences. In line with our hypothesis, we found that delusional ideation and hallucinatory experiences of healthy individuals were predicted by a low resilience against irrelevant information in a probabilistic reasoning task.

In order to quantify the resilience against irrelevant information, we applied a novel computational learning model that allowed for a non-linear relationship between prediction error and learning. Compared to a linear relationship, individuals with a non-linear processing of the prediction error are relatively resilient against an overestimation of excessively surprising information, since the relationship between prediction error and learning is flattened for high prediction errors. On the other hand, they are still capable of rapidly building predictive beliefs about the world, since learning of moderately surprising information (low prediction errors) is not relevantly impaired. By this approach, we were thus able to disentangle inter-individual differences in the *general* speed of learning (that is captured in the learning rate) from specific differences in the impact of former beliefs on learning (that is captured in the resilience against irrelevant information). Our results suggest that specifically the latter is related to an increased proneness for delusional and hallucinatory experiences. Considering that every incoming signal is noisy and naturally contains both relevant and irrelevant information, it seems plausible that an attenuation of specifically the excessively surprising and hence irrelevant information constitutes an effective protection against overhasty and erroneous inferences, while a weakening of this attenuation in turn predisposes for delusional beliefs and hallucinatory percepts. This understanding is additionally supported by our finding that the resilience against irrelevant information indeed was the parameter with the strongest association with hasty inferences (jumping-to-conclusions) and that jumping-to-conclusions was, consistent with prior studies [11,31], in turn related to the proneness for delusional convictions. Obviously, the adaptiveness of a non-linear prediction error processing depends on the particular task: In volatile tasks with frequent changes of the underlying probabilities, large prediction errors might in fact provide vital information, namely that the context of learning has changed. Our task however included no volatility in that sense, because in one round, there were no changes in the task probabilities (the lake, from which fishes were being angled remained the same). Thus, the degree of non-linearity indeed provides a measure for adaptive learning in the adapted beads task.

The idea that a core alteration in psychosis lies in the weighting of new information with regard to prior beliefs has a longstanding history in cognitive schizophrenia research evolving from the suggestion that “the basic disturbance in schizophrenia is 'a weakening of the influences of stored memories of regularities of previous input on current perception‴ [32] via hypotheses of an aberrant attribution of salience to stimuli [8,33] to predictive coding frameworks that embed these hypotheses into a broader framework of Bayesian information processing in the brain [2,3]. In line with this, schizophrenia and/or psychotic symptoms have been consistently linked to the aberrant attribution of salience to stimuli ([34,35] for reviews). Similarly, schizophrenia and psychotic symptoms have been associated with a decreased influence of prior beliefs in perceptual inference ([36], see [37] for a review on visual illusions), although recent work suggests a complex interplay between prior beliefs and perceptual inference in psychosis-related conditions [38,39]. By the use of a feasible and interpretable model, our current study provides a formal description for the interaction between prior beliefs and new information, thereby elucidating the computational mechanisms underlying maladaptive learning and inference in psychosis.

Intriguingly, we found that our participants showed without exception a resilience against irrelevant information that cannot be captured in models that assume a linearly processed prediction error. Especially considering the significant clustering of parameter values in a region with a marked non-linearity in our experiment, this finding suggests that learning in some tasks might not be driven linearly by prediction errors. From a more general perspective, a growing non-linearity between prediction error and learning implies that the impact that a certain new information has on the belief (i.e., the learning) becomes increasingly *independent* from the current belief itself: Whilst under the assumption of a linearly processed prediction error, one and the same information (e.g., a certain fish in our task) has a massively different impact on the learning depending on whether it is surprising or not, a strong non-linearity implies that every fish is treated more or less equally, regardless of the current belief. Similar concepts have previously been proposed in terms of precision-weighted prediction errors, where the learning of strongly surprising information is attenuated if a marked and precise opposing belief has already been built, e.g., if the precision of the belief is high and the precision of the new information low [19]. Compared to these frameworks, our approach has the advantage of simplicity and that the degree of resilience against irrelevant information is captured in one single and easily interpretable parameter: It is noteworthy, that a reduced non-linearity of the relationship between prediction error and learning that could be proven to be associated with psychosis-proneness in this study effectively and straightforwardly models what has been theoretically proposed as a core alteration behind psychotic experiences, namely “a reduction in the precision of prior beliefs, relative to sensory evidence” [1]. Nevertheless, whilst providing a substantial model fit in rather simple tasks like the one carried out in this study, it is questionable if our non-hierarchical model can sufficiently account for more complex environments (e.g., environments with changing volatility). Based on the continuity view of psychosis, we studied psychotic experiences in a sample of non-clinical participants. Mounting evidence suggests that clinical and non-clinical psychotic experiences reflect different expressions of a continuously distributed trait, as they share a common factor structure [40], similar risk factors and demographics [21] as well as a co-clustering in relatives [41,42]. It could moreover be prospectively demonstrated that an increased, but non-clinical proneness for psychotic experiences massively increases the risk of developing a "full" clinical psychosis in the future [43–45], further indicating that non-clinical and clinical psychotic experiences can be explained in terms of similar underlying mechanisms. Importantly, studying psychotic experiences in non-clinical participants does preclude potential confounds associated with clinical diseases and their pharmacological treatment. Hence, although future work is needed to confirm whether our current findings generalize to patients suffering from psychotic disease, the link between maladaptive learning and psychotic experiences established here might generally shed light on the computational mechanisms underlying both non-clinical psychotic experiences and psychosis.

One of the studies limitations is that we only yielded a modest and non-significant association between conventional JTC measures (draws to decision) and psychosis proneness. This is however consistent with previous reports on the relationship between JTC and psychosis proneness in healthy individuals that yielded small effects and mixed results [46–49] and suggest that conventional JTC measures such as draws to decision might not provide a sufficiently fine-grained measure for individual psychosis-related differences in learning and reasoning in healthy individuals.

Taken together, our current findings suggest that a less non-linear processing of prediction error gives rise to overhasty and erroneous inferences, thereby leading to delusional ideas and hallucinatory experiences. Our current work thus empirically substantiates theories that link maladaptive learning to psychotic experiences both in health and disease.

## Supporting Information

S1 Text Mathematical proof that the belief in the non-linear model is bounded between zero and one. (PDF)
