# Correction: Psychotic Experiences and Overhasty Inferences Are Related to Maladaptive Learning

**DOI:** 10.1371/journal.pcbi.1005393

**Published:** 2017-02-17

**Authors:** Heiner Stuke, Hannes Stuke, Veith Andreas Weilnhammer, Katharina Schmack

There is an error in reference 12. The correct reference is: Ross RM, McKay R, Coltheart M, Langdon R (2015) Jumping to conclusions about the beads task? A meta-analysis of delusional ideation and data-gathering. Schizophr Bull 41: 1183–1191. doi:10.1093/schbul/sbu187 PMID: 25616503.
