# Supplementary material for: Psychotic Experiences and Overhasty Inferences Are Related to Maladaptive Learning
Source: PLoS Comput Biol. 2017 Jan 20;13(1):e1005328. doi: 10.1371/journal.pcbi.1005328 (PMC5249047; doi:10.1371/journal.pcbi.1005328)
Supplement: S1 Text — (PDF) [file pcbi.1005328.s001.pdf]

Supplementary Material

Proof that the belief in the non-linear model is bounded between zero and one.

The belief update of our model is defined as follows

$$b_i = f(b_{i-1}, o_i) := b_{i-1} \pm \frac{\alpha}{\zeta + \frac{1}{|b_{i-1} - o_i|}},$$

with $0 \leq \alpha \leq 1$, $0 \leq \zeta$, $0 \leq b_{i-1} \leq 1$ and $o_i = 0, 1$. Furthermore we define $f(x, x) = x$.

We want to show that our model does not produce overshooting beliefs, thus, the belief b_i should be confined to values between zero and one. This means, that f must map the unit interval $I = [0, 1]$ into itself or

$$f(I, o) \subset I, \qquad o \in \{0, 1\},$$

By definition of f we need to check $f(I, 1) \leq 1$, that is

$$x + \frac{\alpha}{\zeta + \frac{1}{1-x}} \leq 1, \qquad x \in [0, 1),$$

We set $y = 1 - x$ and obtain after multiplication with the denominator the following inequality

$$\alpha \leq y\zeta + 1. \tag{1}$$

Because $\alpha \leq 1$ and $0 \leq \zeta$ inequality (1) is satisfied if $0 \leq y$. This is true since $x < 1$.

The proof of $0 \leq f(I, 0)$ is similar.

$$0 \leq x - \frac{\alpha}{\zeta + \frac{1}{x}}, \qquad 0 < x \leq 1,$$

Setting $y = x$ yields inequality (1).
